# One-Step Formation of Pickering Double Emulsion Costabilized by Hydrophobic Silica Nanoparticles and Sodium Alginate

**DOI:** 10.1021/acs.langmuir.4c00976

**Published:** 2024-06-26

**Authors:** Yunxing Li, Jiaming Li, Zhiqing Cai, Yajuan Sun, Hang Jiang, Xin Guan, To Ngai

**Affiliations:** † Key Laboratory of Synthetic and Biological Colloids, Ministry of Education, School of Chemical and Material Engineering, Jiangnan University, Wuxi 214122, P.R. China; ‡ Department of Chemistry, The Chinese University of Hong Kong, Shatin, N. T., Hong Kong, P.R. China

## Abstract

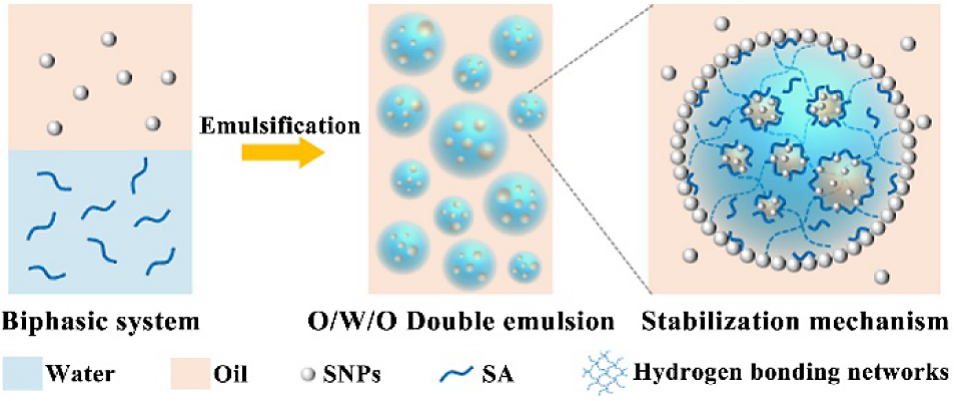

Pickering double emulsions exhibit higher stability and biocompatibility compared with surfactant-stabilized double emulsions. However, tailored synthesis of particle stabilizers with appropriate wettability is time consuming and complicated and usually limits their large-scale adoption. Using binary stabilizers may be a simple and scalable strategy for Pickering double emulsion formation. Herein, commercially available hydrophobic silica nanoparticles (SNPs) and sodium alginate (SA) as binary stabilizers are used to prepare O/W/O Pickering double emulsions in one-step emulsification. The influence of system composition on double emulsion preparation is identified by optical microscopy, confocal laser scanning microscopy, and interfacial tension and water contact angle analyses. The formation of the O/W/O Pickering double emulsion depends critically on the aqueous phase viscosity and occurrence of emulsion inversion. Both hydrophobic SNPs and SA adsorb at the droplet surface to provide a steric barrier, while SA also reduces interfacial tension and increases aqueous phase viscosity, giving double emulsion long-term stability. Their microstructure and stability are controlled by adjusting the SA concentration, water–oil volume ratio, concentration and wettability of the particle stabilizer, and oil type. As a demonstration, the middle layer of the as-prepared O/W/O Pickering double emulsions can be cross-linked in situ with calcium ions to produce calcium alginate porous microspheres. We believe that our strategy for double emulsion formation holds great potential for practical applications in food, cosmetics, or pharmaceuticals.

## Introduction

Double emulsions have attracted significant interest from a variety of disciplines and industries, because of their ability to encapsulate and regulate the release of both water-soluble and oil-soluble cargos and to serve as a template to prepare porous microspheres or microcapsules.^1−11^ Traditionally, water-in-oil-in-water (W/O/W) and oil-in-water-in-oil (O/W/O) are two forms of double emulsions that can be prepared by combining lipophilic and hydrophilic surfactants.^12,13^ Nevertheless, these emulsions are unstable, and questions have been raised regarding their biosafety, which challenge their preparation and applications in various fields.^14−16^ Colloidal particles have been employed to prepare Pickering double emulsions, which improve emulsion stability and avoid use of harmful surfactants.^17−22^ However, the lack of a straightforward and scalable approach for preparing Pickering double emulsions hinders their practical applications.

Pickering double emulsions can be prepared using one of two methods: one-step or two-step emulsification. Usually, two-step emulsification entails the sequential formation of primary and secondary emulsions employing two types of stabilizers, which effectively regulates the emulsion microstructure and encapsulates different cargos.^23,24^ However, it is time consuming and destroys the structure of the primary emulsion during the second emulsification, and therefore, flexibility in industrial large-scale production is lost. Due to its ease of use and effectiveness, one-step emulsification has recently attracted increasing attention for preparing Pickering double emulsions in situations near phase inversion.^17^ Typically, this emulsification uses a single particle stabilizer, mainly including inorganic particles and synthetic polymer particles (micelles, microgel, and latex), with either intermediate hydrophobicity or a distribution of wettability.^25−31^ For example, Bai et al. prepared O/W/O Pickering double emulsions using palmitoyl chloride to increase the hydrophobicity of diatomite particles.^25^ A W/O/W Pickering double emulsion with amphiphilic Janus particles that have a hydrophilic area rich in acrylic acid and a hydrophobic area rich in styrene was described by Lee et al.^26^ Nonomura et al. found that silicone particles shaped like a bowl with varying wettability caused by contact angle hysteresis due to solvent entrapment can stabilize Pickering double emulsions.^27^ Lin et al. first synthesized block copolymer micelles with predesigned hydrophilic and hydrophobic block lengths and used them to prepare Pickering double emulsions.^30^ However, particle stabilizers discussed in these research studies usually require particular surface modifications or tailored design and synthesis, which complicates the double emulsion preparation and compromises the large-scale adoption of particle stabilizers.

Microfluidic emulsification has also been developed that uses various flow focusing techniques to prepare double emulsions with high monodispersity in a one-step process, which is more appropriate for surfactant-based systems.^31^ There have been few studies on the preparation of Pickering double emulsions using a microfluidic platform. For example, two different particle stabilizers, such as tetra(ethylene glycol)-functionalized gold nanoparticles and CdSe quantum dots, have been used to stabilize two interfaces of Pickering double emulsions.^21^ Although microfluidic emulsification allows for the controllable and efficient preparation of double emulsions, it often has a low production yield. The scaling up of microfluidic-based approaches remains a great challenge. To achieve the simpler and more efficient preparation of Pickering double emulsions in a one-step process, recently, an alternative strategy has been reported using binary stabilizers. This also makes it possible to prepare Pickering double emulsions on a large scale. Our group found that a stable O/W/O Pickering double emulsion could be generated by one-step emulsification, thanks to hydrophobic poly(*N*-isopropylacrylamide) microgels and hydrophilic lipase that stabilize two interfaces of opposing curvature.^32^ Nonetheless, little research has been done on how binary stabilizers in a simple formulation affect Pickering double emulsion formation, especially for applications in cosmetics, food, or pharmaceuticals. Therefore, the main focus here is on how Pickering double emulsions with improved stability, biocompatibility, and biodegradability are formed using binary stabilizers.

Herein, a one-step emulsification is employed to generate O/W/O Pickering double emulsions via phase inversion using commercially available hydrophobic silica nanoparticles (SNPs) and water-soluble sodium alginate (SA). Hydrophobic SNPs and SA can adsorb at both oil–water interfaces of opposing curvatures, preserving the structural integrity of the emulsion droplets. The high-viscosity SA solution captures small oil droplets dispersed in large water droplets during emulsion inversion, limiting their movement and collision during storage, which is beneficial for the formation of Pickering double emulsions and their long-term stability. To verify the versatility of our strategy, a series of factors influencing double emulsion preparation, including SA concentration, volume ratio of water to oil, concentration and wettability of SNPs, and oil type, were identified. Finally, the application of the prepared double emulsions as templates for preparing porous microspheres was preliminarily explored.

## Materials and Methods

### Materials

Hydrophobic silica nanoparticles (R816, R974, R812(s), and R202) and hydrophilic silica nanoparticles (A200) were kindly provided by Evonik (Germany). According to Evonik’s claim, the hydrophobicity of R816, R974, R812(s), and R202 increases from left to right. Unless otherwise stated, hydrophobic silica nanoparticles (SNPs) usually refer to R974. Sodium alginate (SA) was supplied by Shandong Jiejing Corporation (China). Sunflower oil, rhodamine B (RhB), EDTA-Ca, and castor oil were provided by Macklin Biochemical Co., Ltd. (China). Croda (UK) supplied the caprylic/capric triglycerides (GTCC). Cyclopentasiloxane (D5) was bought from ShinEtsu (Japan). d-Gluconic acid-δ-lactone (GDL) was bought from InnoChem Science & Technology Co., Ltd. (China). Isohexadecane was provided by Sinopharm Chemical Reagent Co., Ltd. (China). Nile Red was supplied by Merck (Germany). All of the experiments were conducted using deionized water.

### Preparation of Aqueous Solution of SA

Aqueous solutions of SA at various concentrations (0.5% to 5%, w/v) were prepared by adding different amounts of SA powder to a specified volume of deionized water, and the mixture was stirred mechanically for 8 h. Before emulsification, it was left under ambient conditions for enough time in order to allow any bubbles during mixing to disappear.

### Preparation of Emulsions Stabilized by Hydrophobic SNPs or SA

Hydrophobic SNPs (1%, w/v) were first dispersed in GTCC under vortex and ultrasound. After mixing with an equal volume of water, the mixture was homogenized at 16 000 rpm for 2 min to obtain an emulsion. As for SA, GTCC (3 mL) was combined with an equal volume of the SA aqueous solution (2.5%, w/v). The emulsion was then obtained by homogenizing the above mixture at 16 000 rpm for 2 min.

### Preparation of Emulsions Stabilized by Both SNPs and SA

First, a certain amount of SNPs was dispersed in GTCC (3 mL) under vortex and ultrasound, followed by the addition of an equal volume of SA aqueous solution at varying concentrations. The aforesaid mixture was homogenized at 16 000 rpm for 2 min to prepare emulsions. In order to study how various parameters affect the emulsion preparation, hydrophobic SNPs of varying degrees and hydrophilic SNPs were examined. The same volumes of isohexadecane, D5, sunflower oil, and castor oil were also used instead of GTCC. Furthermore, the influence of water–oil volume ratios (8:1 to 1:8) was examined using the same emulsification process. The total emulsion volume was maintained at 6 mL for all samples, which were homogenized at 16 000 rpm for 2 min. The concentration of R974 was 1 % in weight of particle/volume of oil phase, while the concentration of SA was 2.5 % in weight of polymer/volume of the aqueous phase.

### Preparation of Calcium Alginate Porous Microspheres

Hydrophobic SNPs (1%, w/v) were first dispersed in GTCC. After mixing with an equal volume of water dissolved with SA (3%, w/v), EDTA-Ca (2%, w/v), and GDL (2%, w/v), the mixture was homogenized at 16 000 rpm for 2 min to obtain an emulsion. After being left for 2 h, the resultant microspheres were collected by centrifugation, and then, they were washed with isopropanol three times.

### Characterization

The emulsion microstructure was examined using a Leica DM500 optical microscope (Germany) and a TCS SP8 Leica confocal laser scanning microscope (Germany) at an excitation wavelength of 530 nm, respectively. Nile Red was used to stain the oil, and the particle stabilizer was labeled with RhB. RhB dissolved in an aqueous phase could adsorb to the barrier layer of hydrophobic SNPs formed at an oil/water interface.^33^ The Dataphysics OCA15EC (Germany) with an image-capturing device was used to perform the contact angle measurements. A sufficient quantity of particle powder was pressed into a disk, and then, water droplets with various concentrations of SA were dropped on its surface in air. The same instrument was also used to record the dynamic interfacial tensions of the water/GTCC with or without various additional components over time. The viscosity of a series of SA aqueous solutions was measured using an IKA ROTAVISC ME-VI viscometer (Germany) at room temperature coupled with different T-bar type spindles at 20 to 200 rpm. The morphology of porous microspheres was observed with an S-4800 scanning electron microscope (Hitachi, Japan). The sample was coated with a gold overlayer before examination.

## Results and Discussion

### Formation and Stabilization of O/W/O Pickering Double Emulsion

**Figure 1:**
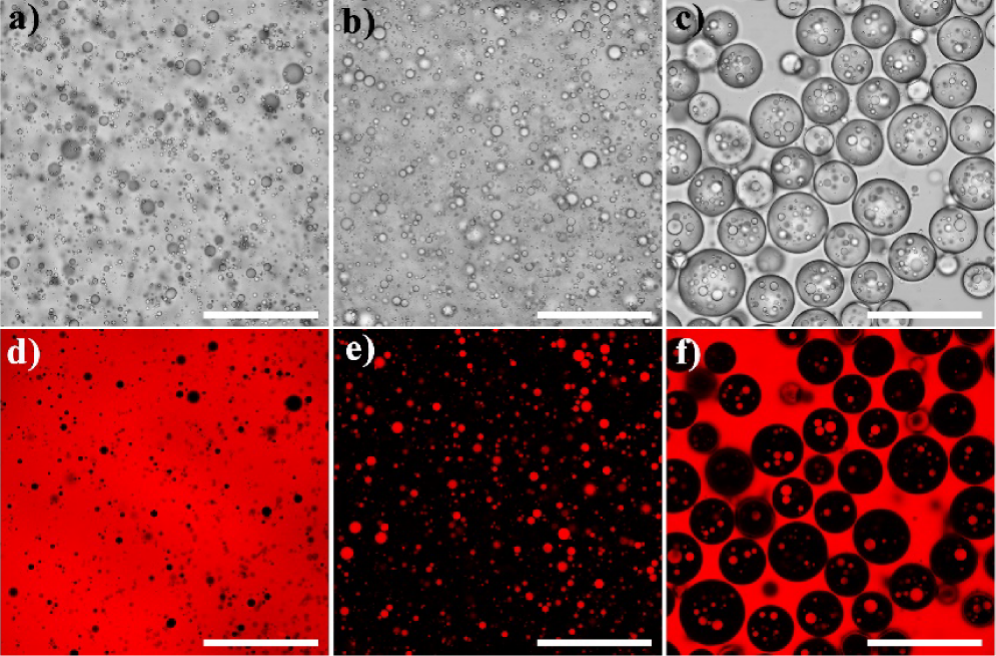
Optical microscopy and CLSM images of emulsions prepared with 1% hydrophobic SNPs (a,d), 2.5% SA (b,e), or 1% hydrophobic SNPs and 2.5% SA (c,f). Scale bars are 200 μm.

Prior to the preparation of emulsions with two stabilizers simultaneously, the formation of emulsions with either water-soluble SA or hydrophobic SNPs alone was investigated with GTCC as the oil phase. Figure 1a shows that a typical W/O Pickering single emulsion with a small droplet size was obtained using hydrophobic SNPs as stabilizers (Figure 1a,d). Its average droplet size, according to statistical analysis, is about 7.21 μm (Figure S1a). In comparison, SA, as a biopolymer, is able to stabilize an O/W single emulsion with an average droplet size of about 8.96 μm (Figures 1b,e and S1b). When both hydrophobic SNPs and SA were used, notably, an O/W/O Pickering double emulsion, i.e., many smaller oil droplets enclosed inside one big water droplet, could be successfully prepared (Figure 1c,f). It can be observed that the water droplet size is substantially larger than that of Pickering single emulsion. Statistical results indicate that the average droplet size of big water droplets is approximately 57.54 μm (Figure S1c). Interestingly, there is less variation in the average size of inner oil (Figure S1d, ca. 9.23 μm) compared to the SA-stabilized single emulsion, implying that they are primarily stabilized by SA.

**Figure 2:**
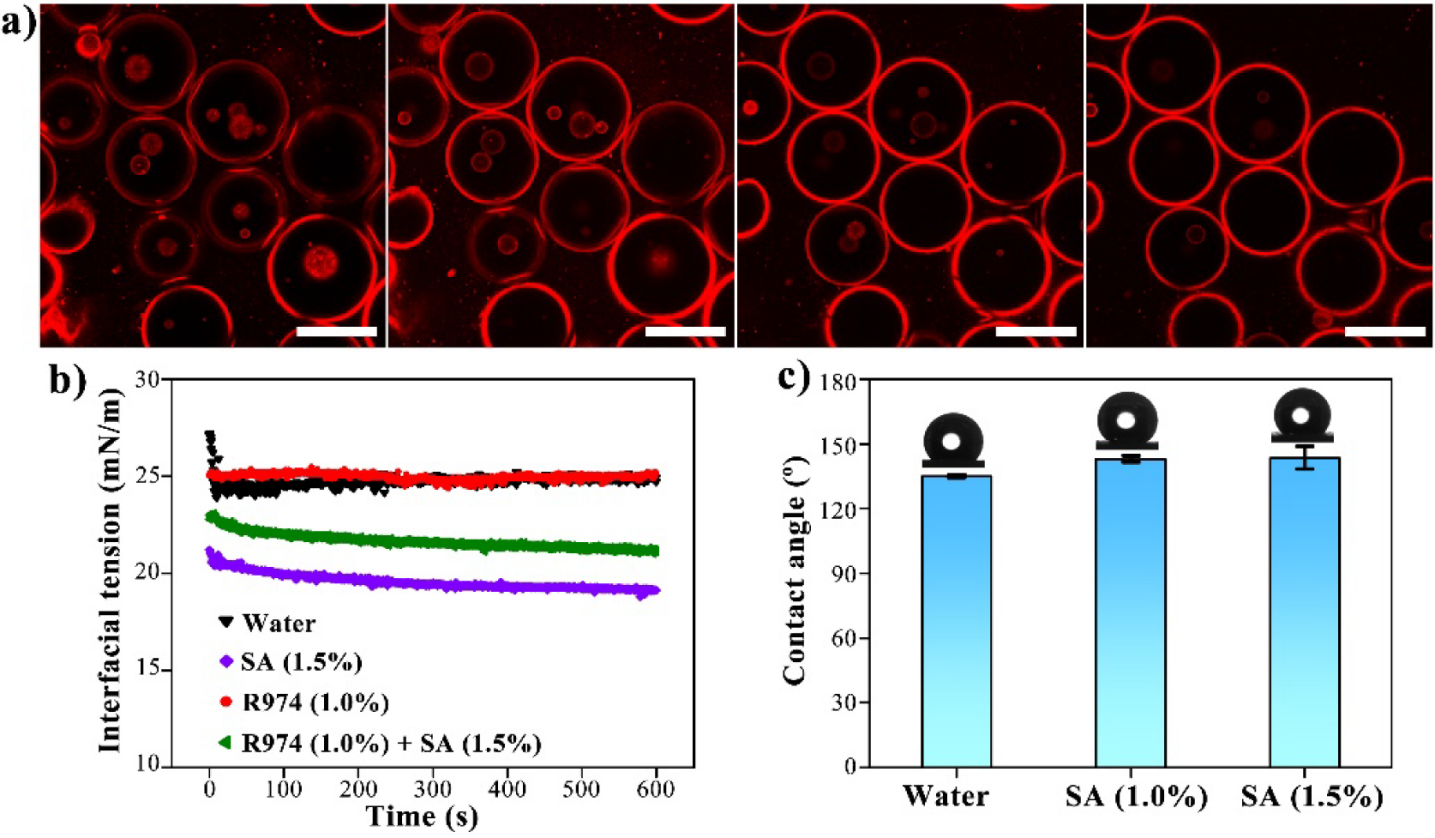
(a) CLSM images at various focus depths of the emulsion prepared with 1% RhB-labeled hydrophobic SNPs and 2.5% SA. Scale bars are 50 μm. (b) Dynamic interfacial tensions of the interface of water/GTCC with different components. (c) Contact angles of a water drop at various SA concentrations on the pellets composed of hydrophobic SNPs in air.

CLSM was used to examine the interfacial microstructure of the prepared double emulsion in order to validate our hypothesis. From Figure 2a, the adsorbed layer generated by the accumulation of RhB-stained hydrophobic SNPs between water droplets reveals the type and stabilization of the prepared double emulsions. Interestingly, partial SNPs are found inside the inner oil droplets, which could be due to hydrophobic SNPs uniformly dispersed in the oil phase before emulsification. Figure 2b shows that SA could decrease the interfacial tension to a lower value, whereas hydrophobic SNPs have poor interfacial activity and are unable to do so. By combination of these two stabilizers in one system, the adsorption of SA at the interface is inhibited. This is feasible because the hydrophobic SNPs occupy a portion of the interface area, leaving less space for SA to attach. In accordance with the W/O single emulsion prepared with hydrophobic SNPs alone, from Figure 2c, water contact angle measurement reveals that they are hydrophobic. Because of the interfacial curvature, hydrophobic SNPs are better suited to stabilize the water-in-oil interface, which is consistent with the CLSM results. In addition, the contact angle of SA aqueous solution on the hydrophobic SNP tablet do not vary much, suggesting that there may not be a substantial interaction between the hydrophobic SNPs and SA. In other words, the hydrophobic SNPs and SA could coassemble at the oil–water interface of different curvatures, but SA is mostly responsible for stabilizing the inner oil droplets, while the hydrophobic SNPs are primarily responsible for stabilizing the outer water droplets.

### Effect of SA Concentration on Emulsion Formation and Stability

**Figure 3:**
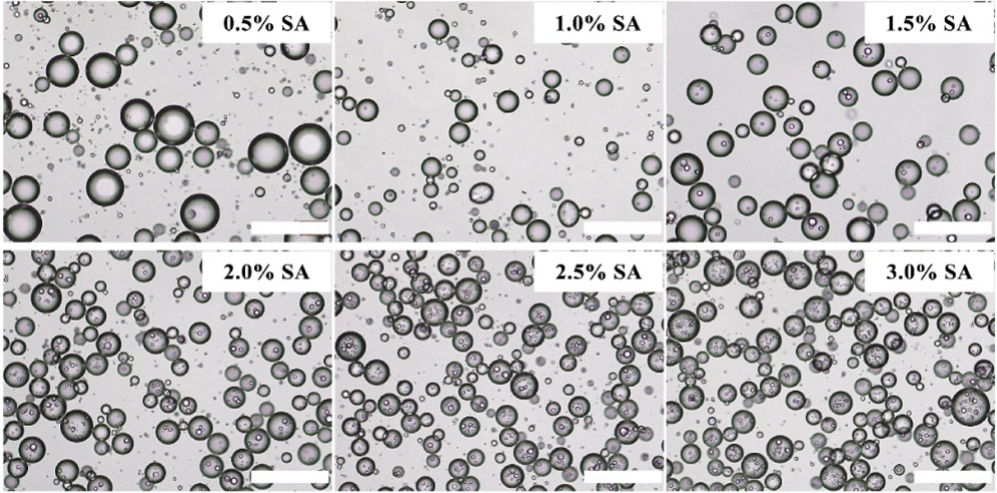
Optical microscopy images of emulsions prepared with 1% hydrophobic SNPs and various SA concentrations. In each optical image, the scale bar is 200 μm.

The emulsion preparation was then investigated in terms of the SA concentration. From Figure 3, it is worth noting that almost only single emulsions occur at low SA concentrations (1%). When the SA concentration is increased to over 1.5%, the Pickering double emulsions are formed. The average size of outer water droplets decreases from 57.92 to 52.45 μm, when the SA concentration is increased from 1.5% to 3% (Figure S2). Moreover, more and smaller inner oil droplets are generated in these double emulsions with higher SA concentrations. Because SA molecules have −OH and −COOH groups, there may be intramolecular and intermolecular hydrogen bonding within and between them.^34^ Consequently, the viscosity of the aqueous phase can be effectively increased by SA-induced hydrogen bonding networks, offering better resistance to the coalescence of both inner and outer droplets as well as escape of the inner droplet into the outer continuous phase. A previous study found the oil viscosity reaching a high value was to obtain the W/O/W Pickering double emulsions using silica particles modified by amino silane and SA simultaneously; otherwise, it cannot be obtained.^35^ As a result, SA and hydrophobic SNPs, respectively, are the primary factors determining the stability of inner and outer droplets because of their oppositely intrinsic wettability. Moreover, the SA-induced polymer networks that result in a high viscosity of the aqueous phase also contribute to the stabilization of prepared double emulsion.

**Figure 4:**
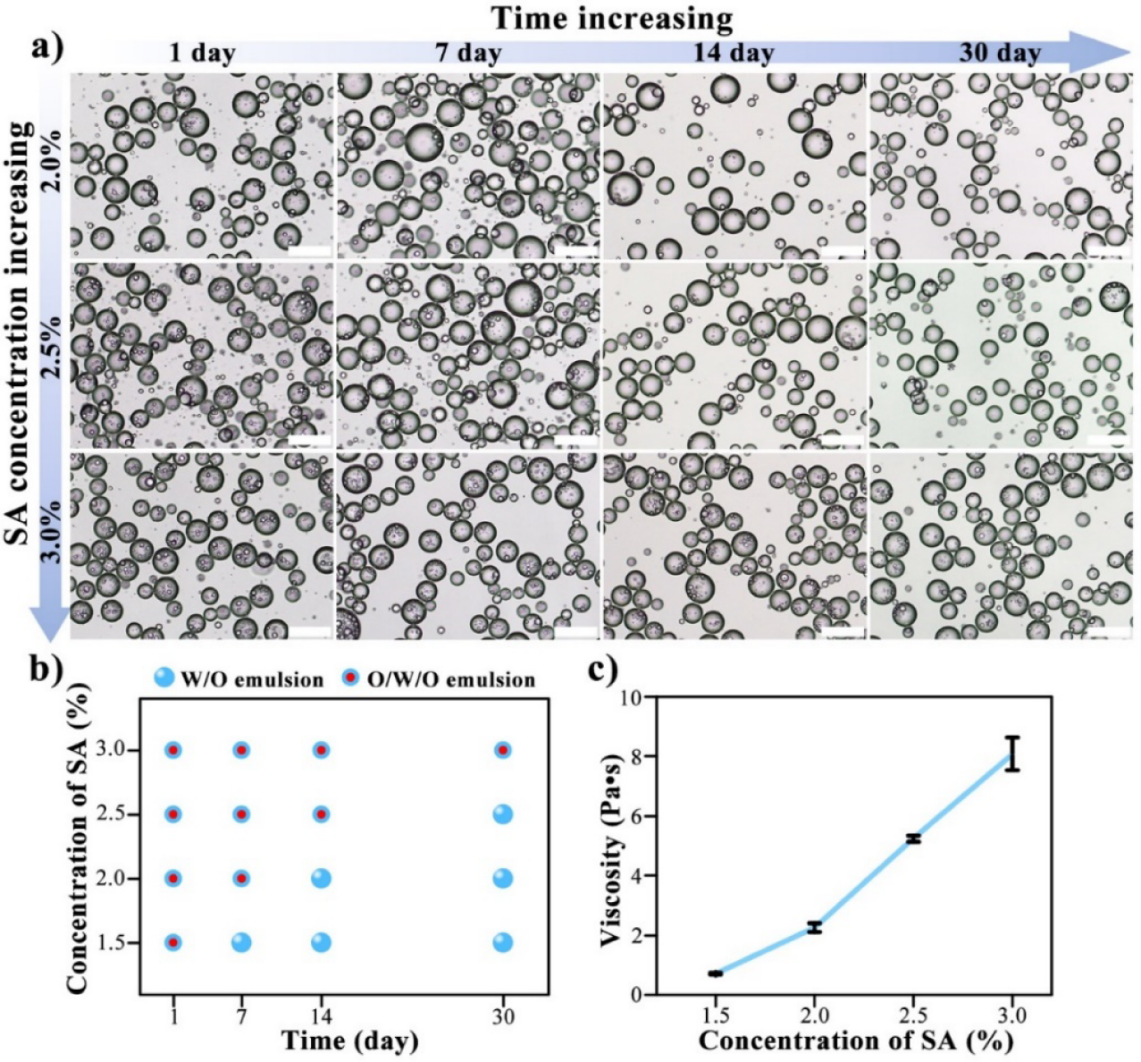
(a) Optical microscopy images of emulsions prepared with 1% hydrophobic SNPs and increasing concentrations of SA at room temperature; (b) variation in the type of emulsions prepared with 1% hydrophobic SNPs and various SA concentrations under storage at room temperature; (c) viscosity of the aqueous solutions of SA at a series of concentrations. Scale bars are 100 μm.

Notably, the viscosity of the aqueous phase not only facilitates the formation of Pickering double emulsions but also affects their long-term stability. The findings of 30 days of recording the emulsion microstructure indicate that Pickering double emulsions with 1.5% SA could only maintain their stability for 1 day, whereas the stability can be effectively sustained at the SA concentrations over 2.5% (Figure 4a,b). At a 3% SA concentration, the Pickering double emulsion remains stable for a minimum of one month. In line with the variation in appearance and outer water droplet size distributions over 1 month (Figures S3 and S4), the instability of these emulsions is mostly caused by the disappearance of inner oil droplets. Figure 4c shows that the aqueous phase with a higher SA concentration has a higher viscosity. In line with the results in Figure 4a,b, Pickering double emulsions are only generated when the aqueous phase viscosity ranges from about 0.70 to 8.1 Pa·s, and the higher the viscosity, the slower the double emulsions disappear. However, too high viscosity can limit the flow and dispersion of the aqueous phase, making the preparation of double emulsions inefficient. As shown in Figure S5, when the SA concentration rises above 4%, the proportion of Pickering double emulsion droplets in the resulting emulsion dramatically decreases.

### Effect of the Volume Ratio of Water to Oil on Emulsion Formation

**Figure 5:**
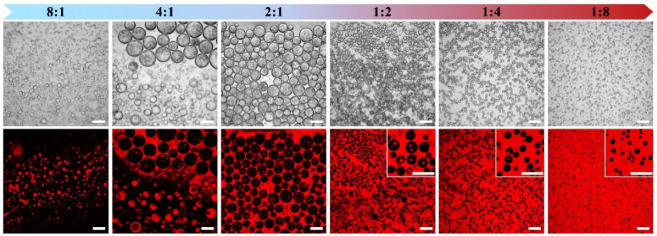
Optical microscopy and CLSM images of emulsions prepared with 1% hydrophobic SNPs and 2.5% SA at various water/oil volume ratios. Images and insets have scale bars of 200 and 100 μm, respectively.

CLSM images clearly reveal that catastrophic phase inversion occurs when the water/oil volume ratios vary from 8:1 to 1:8, resulting in emulsion inversion from O/W to W/O (Figure 5). At a 4:1 water to oil volume ratio, the W/O Pickering single emulsion and O/W/O Pickering double emulsion are present concurrently. At intermediate water/oil volume ratios ranging from 2:1 to 1:4, only O/W/O Pickering double emulsions are obtained. In addition, as the volume fraction of aqueous phase decreases, so do the average diameter of water droplets and the quantity of oil droplets embed within them. When the volume ratio of water to oil is decreased below 1:4, a W/O Pickering single emulsion is formed. Recent studies have demonstrated that phase inversion plays a major role in the one-step preparation of double emulsions using a single type of particle or block polymer.^16,26,36,37^ An O/W single emulsion is generated at a low volume ratio of the oil phase due to the high viscosity of the aqueous phase and a certain emulsifying ability of SA. The formation of a W/O Pickering single emulsion is the result of phase inversion brought on by an increase in the volume fraction of the oil phase. During emulsion inversion, the larger water droplets that develop later capture the smaller oil droplets that have formed in water initially. The reasons behind it could be that the mobility of trapped small oil droplets is hindered by the high viscosity of the aqueous phase. Favis et al. have reported a similar phenomenon that the production of subembedded droplets through phase inversion during the processing of binary molten polymers.^38,39^ The subembedded droplet concentration rises as the dispersion phase viscosity increases because its mobility decreases. In addition, the diffusion of inner oil droplets into the external oil continuous phase could be prevented by the steric barrier of particle and biopolymer stabilizers at the interface. The above formation mechanism may also account for the development of a W/O single emulsion at low volume fraction of the aqueous phase, since it lowers the size of water droplets formed via phase inversion, thus reducing the chance of smaller oil droplets embedding.

### Effect of Particle Concentration on Emulsion Preparation at Fixed Hydrophobicity

**Figure 6:**
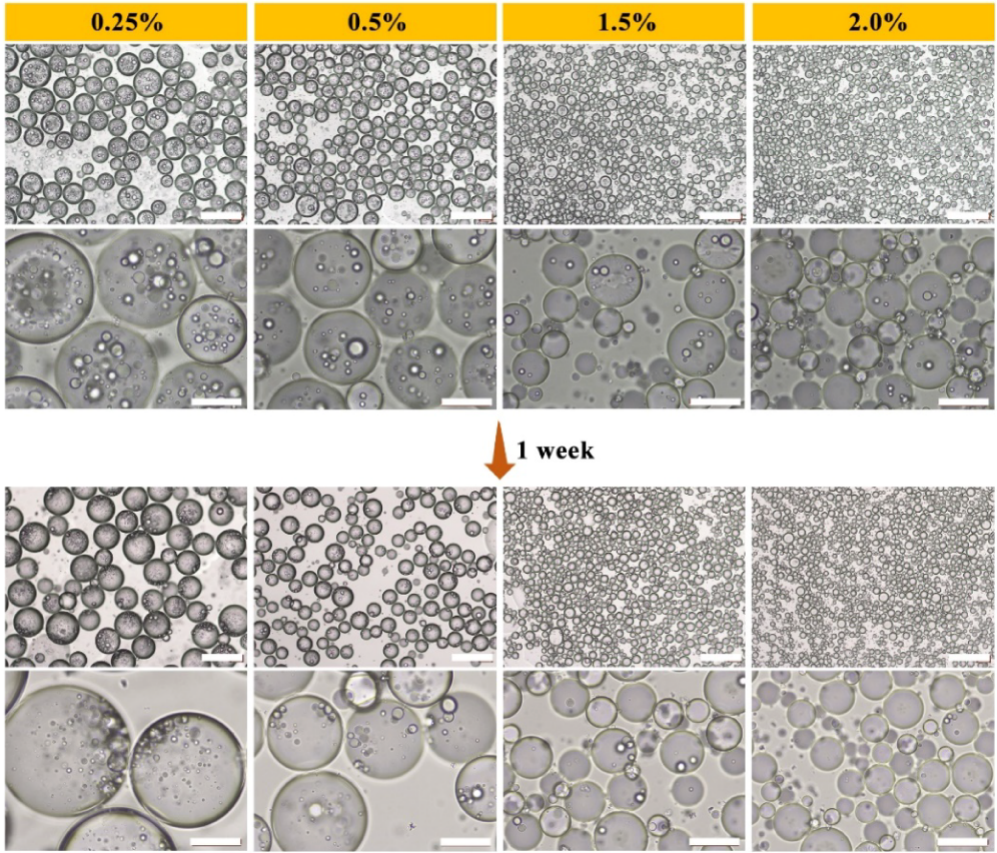
Optical microscopy images of emulsions prepared with 2.5% SA and various concentrations of hydrophobic SNPs during storage at room temperature. Scale bars are 200 μm for the optical microscopy images at low magnification and 50 μm those at high magnification.

The concentration of hydrophobic SNPs is also significant in maintaining the formation and stability of the resulting Pickering double emulsions. From Figures 6 and S6, the outer water droplet size decreases as the concentration of hydrophobic SNPs increases, ranging from 88.57 to 30.19 μm, which also indicates that hydrophobic SNPs play a major stabilizing role in the outer water droplets. Furthermore, the appearance of Pickering double emulsions during storage at room temperature demonstrates that the presence of a higher concentration of hydrophobic SNPs in the oil phase considerably prevents sedimentation (Figure S7). In addition to a decrease in the outer water droplet size, this result is also likely due to the presence of interconnected particle bridges between adjacent outer droplets, resulting in the improved long-term stability of double emulsions.^33^ But it should be highlighted that although a high concentration of hydrophobic SNPs improves the stability of outer water droplets, the formation and stability of inner oil droplets are compromised. Specifically, the lower the concentration of hydrophobic SNPs, the greater the variation in the average size of outer water droplets over time (Figure S6). In addition, when the concentration of hydrophobic SNPs is increased, the average number of smaller inner oil droplets decreases. This could be because as the water droplets formed in phase inversion decrease in size, smaller oil droplets are less likely to be engulfed by them. Furthermore, all oil droplets are expelled to the outer oil phase after 1 week of storage in the presence of 2% hydrophobic SNPs. These findings imply that the stability of inner oil droplets is mostly determined by SA adsorption on the droplet surface. The high concentration of hydrophobic SNPs may inhibit SA adsorption on the surface of the inner oil droplets, resulting in a decrease in their stability. Furthermore, when the outer water droplets shrink, so do the distance at which the encapsulated oil droplets diffuse out, causing the inner oil droplets to disappear faster.

### Effect of Particle Wettability on Emulsion Formation at a Fixed Concentration

**Figure 7:**
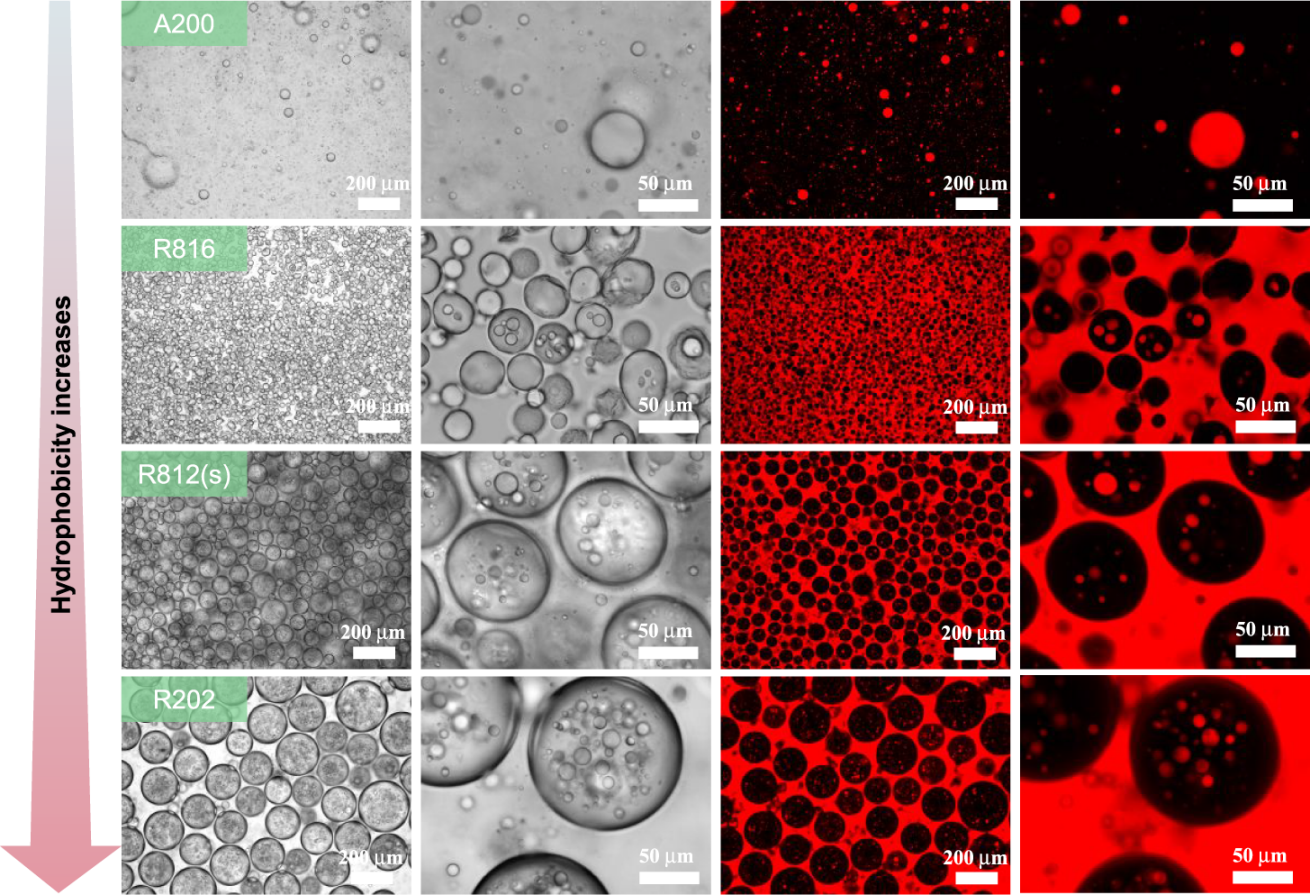
Optical microscopy and CLSM images of emulsions prepared with 2.5% SA and 1% other commercially available SNPs with various hydrophobicity.

The wettability of particle stabilizers influences the Pickering emulsion type. Therefore, we further evaluated the emulsion preparation using other commercially available particles with varying degrees of hydrophobicity. As can be seen in Figure 7, hydrophilic SNPs (A200) can only cooperate with SA to stabilize an O/W single emulsion. However, the formation of O/W/O Pickering double emulsions of varied microstructures is achieved by increasing particle hydrophobicity (R816, R812(s), and R202). In detail, hydrophobic SNPs (R816) with moderate hydrophobicity tend to form double emulsions with a relatively small average size of outer water droplets (Figure S8a, ca. 26.88 μm), with just a portion of them containing several smaller oil droplets. The double emulsion prepared using most hydrophobic SNPs (R202) has the largest size of outer water droplets (Figure S8c, ca. 203.31 μm) with more oil droplets trapped inside. The microstructure of emulsion formed with hydrophobic SNPs (R812(s)) is intermediate between that of the two samples prepared with R816 and R202. Its average droplet size of the outer water droplets is about 82.56 μm (Figure S8b). These results further demonstrate that the hydrophilic SA and hydrophobic nanoparticles mainly adsorb at the surfaces of inner oil droplets and outer water droplets, respectively, allowing the stabilization of Pickering double emulsions. Furthermore, excessively hydrophobic nanoparticles are unsuitable for stabilizing outer water droplets, which lead to an increase in their size, and it makes sense that more oil droplets would get trapped in the larger water droplets during the phase inversion, based on our proposed O/W/O Pickering double emulsion formation mechanism.

### Effect of Oil Type on Emulsion Formation

**Figure 8:**
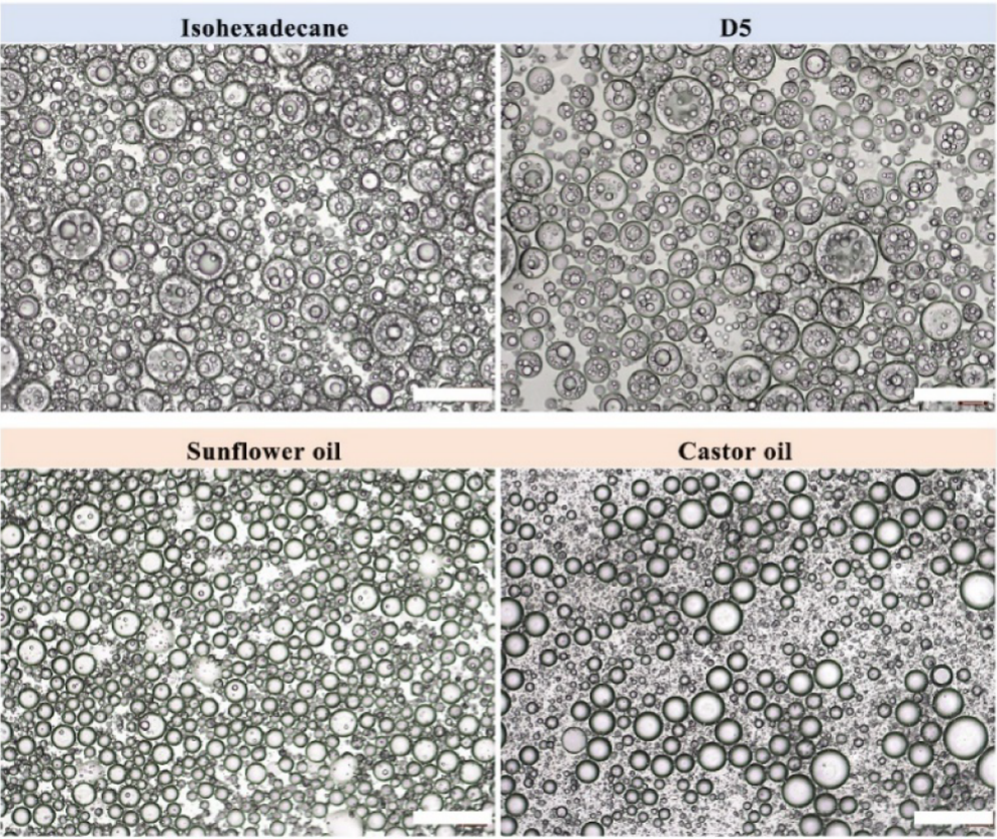
Optical microscopy images of emulsions prepared with 2.5% SA and 1% hydrophobic SNPs while isohexadecane, cyclopentasiloxane (D5), sunflower oil, and castor oil are oil phases, respectively. Scale bars are 200 μm.

Considering that the polarity and composition of oil could affect the emulsion preparations, we investigated the oil phase properties on the development of Pickering double emulsion further (Figure 8).^40,41^ Some oils permitted for use in cosmetics are chosen. In the case of highly pure isohexadecane and D5 with different polarities, the O/W/O Pickering double emulsions are formed. However, when vegetable oils, such as sunflower oil and castor oil, are employed, double emulsions may be considered to be unsuccessfully prepared. Typical W/O Pickering single emulsions are formed. This phenomenon could be attributed to the presence of surface-active substances, which are able to adsorb at the interfaces of oil–water and change both their tensions and curvatures.^41^ Consequently, the encapsulated oil droplets are not stable because the competitive adsorption prevents SA from adhering to the oil–water interface.

### Preparation of Calcium Alginate Porous Microspheres

**Figure 9:**
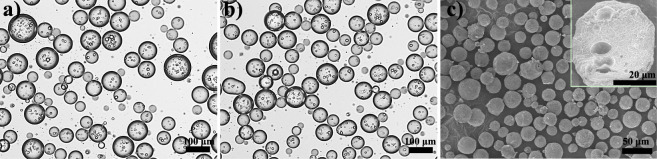
Optical microscopy images of a double emulsion template (a) and calcium alginate porous microspheres (b) formed from it; the SEM image of calcium alginate porous microspheres (c), with an inset of a microsphere cross-section.

For demonstrating the application of our O/W/O Pickering double emulsions, calcium alginate porous microspheres were prepared by cross-linking alginate in situ with Ca^2+^ released from EDTA-Ca via pH reduction using d-gluconic acid-δ-lactone (GDL) hydrolysis.^42^ From Figure 9, the optical images show that the resultant porous microspheres (ca. 68.4 μm) are similar in the average size to the water droplets (ca. 69.6 μm) found in the Pickering double emulsion. The SEM image reveals that these microspheres are quasi-spherical and well dispersed. The significant reduction in the average size of microspheres (ca. 25.5 μm) observed by SEM could be attributed to drying shrinkage prior to examine. Notably, the inset in Figure 9c shows some macrovoids in the cross-section of the microspheres, corroborating their porous structure. Therefore, the O/W/O Pickering double emulsions, which are stabilized by SA and hydrophobic SNPs, work well as templates for preparing porous microspheres. This method has two advantages, including one-step preparation of emulsion templates and biopolymers with good biocompatibility for porous skeletons. These porous microspheres are expected to be used to encapsulate and protect water-soluble drugs as well as for enzyme immobilization.

## Conclusions

In conclusion, we develop a facile yet effective strategy for the one-step preparation of O/W/O Pickering double emulsions via phase inversion by combining water-soluble SA and hydrophobic SNPs. The results indicate that hydrophobic SNPs and SA have an influence on the stability of the outer and inner emulsion droplets, respectively. In addition to conventional emulsion stabilization that relies on the adsorption of the stabilizer at oil–water interfaces, the stability of the as-prepared O/W/O Pickering double emulsions is also affected by the viscosity of the aqueous phase. The high viscosity of the aqueous phase significantly restricts the movement and coalescence of inner oil droplets, resulting in higher double emulsion stability. More importantly, the microstructure and stability of the resulting Pickering double emulsions can be easily regulated by adjusting the system composition including the SA concentration, volume fraction of aqueous phase, concentration and wettability of particle stabilizers, and oil type. Finally, we demonstrate that calcium alginate porous microspheres can be prepared by in situ cross-linking using the O/W/O Pickering double emulsions that are obtained as templates. We believe that our novel strategy, which combines ease of operation, simple formulation, and commercially available ingredients, holds promise for enormous practical applications in cosmetics, food, or pharmaceuticals.

## Supporting Information Available

Macroscopic and microscopic images as well as droplet size distributions of O/W/O Pickering double emulsions prepared with different conditions (PDF)
