# Supplementary material for: One-Step Formation of Pickering Double Emulsion Costabilized by Hydrophobic Silica Nanoparticles and Sodium Alginate
Source: Langmuir. 2024 Jun 26;40(27):13903–11. doi: 10.1021/acs.langmuir.4c00976 (PMC11238577; doi:10.1021/acs.langmuir.4c00976)
Supplement: Supplementary file 1 — la4c00976_si_001.pdf [file la4c00976_si_001.pdf]

Supporting Information

One-step formation of Pickering double emulsion co-stabilized by hydrophobic silica nanoparticles and sodium alginate

Yunxing Li[†*], Jiaming Li[†], Zhiqing Cai[†], Yajuan Sun[†], Hang Jiang[†], Xin Guan[‡], and To Ngai[‡*]

[†] *Key Laboratory of Synthetic and Biological Colloids, Ministry of Education, School of Chemical and Material Engineering, Jiangnan University, Wuxi 214122, P. R. China*

[‡] *Department of Chemistry, The Chinese University of Hong Kong, Shatin, N. T. Hong Kong, P. R. China*

* Corresponding author

Email: yunxingli@jiangnan.edu.cn (Y. X. Li); tongai@cuhk.edu.hk (T. Ngai)

Number of pages: 5

Number of figures: 8

Table of Contents

Figure S1. Droplet size distributions of emulsion prepared with 1% hydrophobic SNPs (a), 2.5% SA (b) or 1% hydrophobic SNPs and 2.5% SA (c and d).

Figure S2. Droplet size distributions of emulsion (outer water droplet) prepared with 1% hydrophobic SNPs and various SA concentrations. (a) 1.5%, (b) 2%, (c) 2.5%, and (d) 3%.

Figure S3. Variation in droplet size distributions of emulsion (outer water droplet) prepared with 1% hydrophobic SNPs and various SA concentrations. (a) 2%, (b) 2.5%, and (c) 3%.

Figure S4. Variation in appearance of emulsion prepared with 1% hydrophobic SNPs and

various SA concentrations.

Figure S5. Optical microscopy images of the O/W/O Pickering double emulsions prepared with 1% hydrophobic SNPs and different concentrations of SA. Scale bars are 200 μm.

Figure S6. Droplet size distributions of Pickering emulsions (outer water droplet) prepared with 2.5% SA and various concentrations of hydrophobic SNPs during storage at room temperature.

Figure S7. Appearance of Pickering emulsions prepared with 2.5% SA and various concentrations of hydrophobic SNPs during storage at room temperature.

Figure S8. Droplet size distributions of emulsion (outer water droplet) prepared with 2.5% SA and 1% other commercially available SNPs with various hydrophobicity. (a) R816, (b) R812(s) and (c) R202.

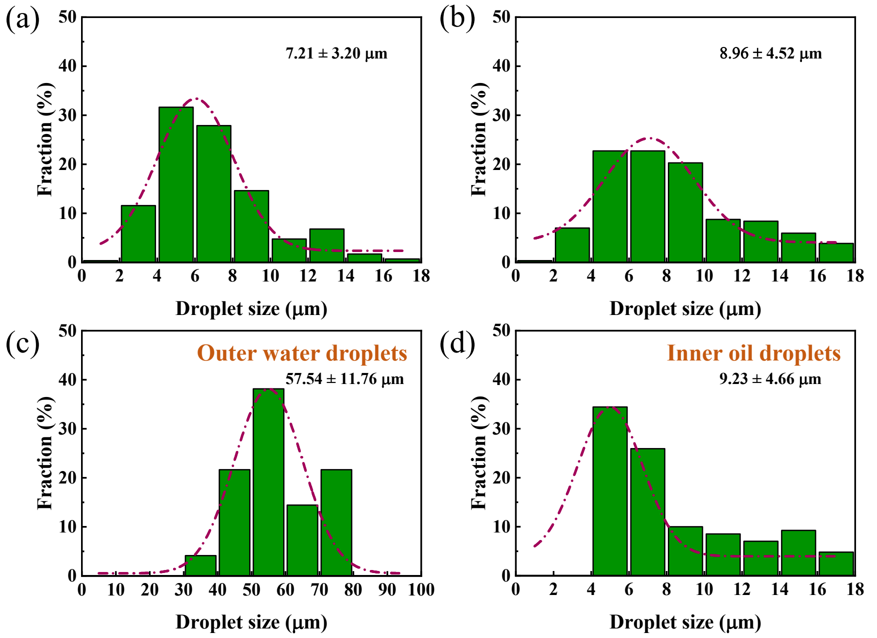

Figure S1. Droplet size distributions of emulsion prepared with 1% hydrophobic SNPs (a), 2.5% SA (b) or 1% hydrophobic SNPs and 2.5% SA (c and d).

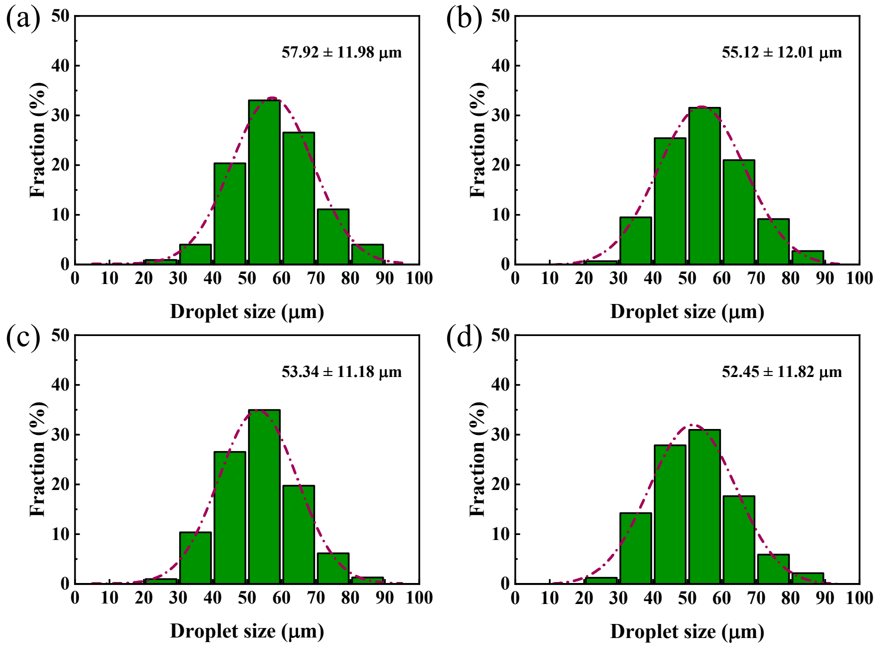

Figure S2. Droplet size distributions of emulsion (outer water droplet) prepared with 1% hydrophobic SNPs and various SA concentrations. (a) 1.5%, (b) 2%, (c) 2.5%, and (d) 3%.

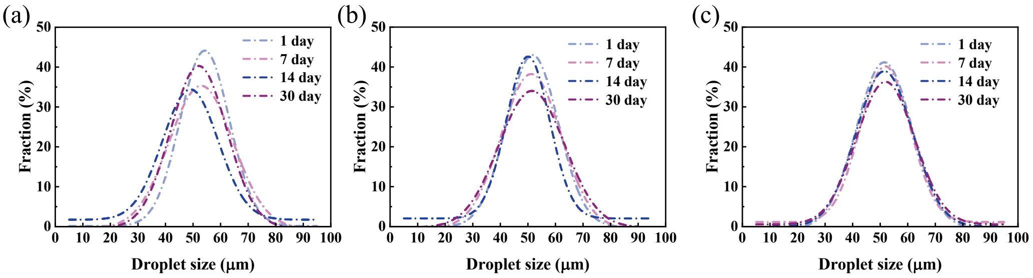

Figure S3. Variation in droplet size distributions of emulsion (outer water droplet) prepared with 1% hydrophobic SNPs and various SA concentrations. (a) 2%, (b) 2.5%, and (c) 3%.

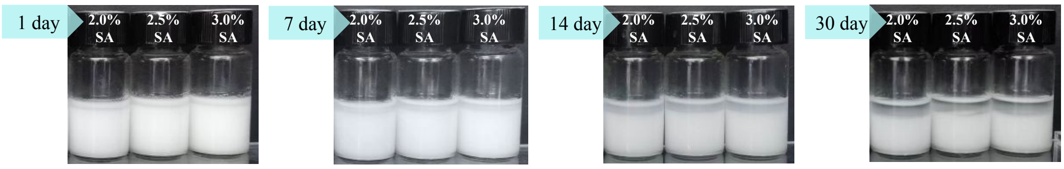

Figure S4. Variation in appearance of emulsion prepared with 1% hydrophobic SNPs and various SA concentrations.

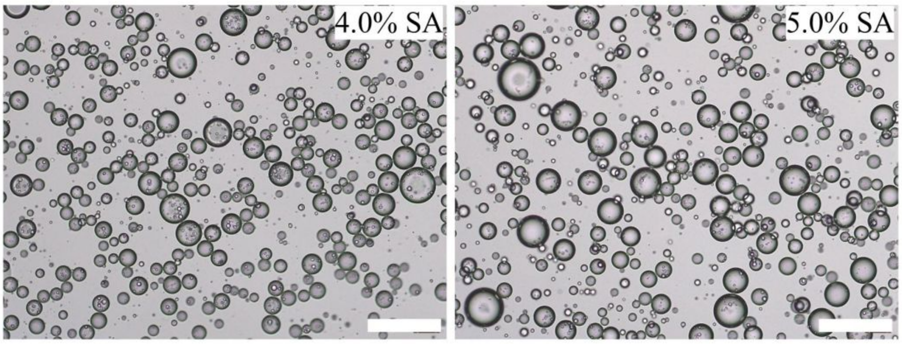

Figure S5. Optical microscopy images of the O/W/O Pickering double emulsions prepared with 1% hydrophobic SNPs and different concentrations of SA. Scale bars are 200 μm.

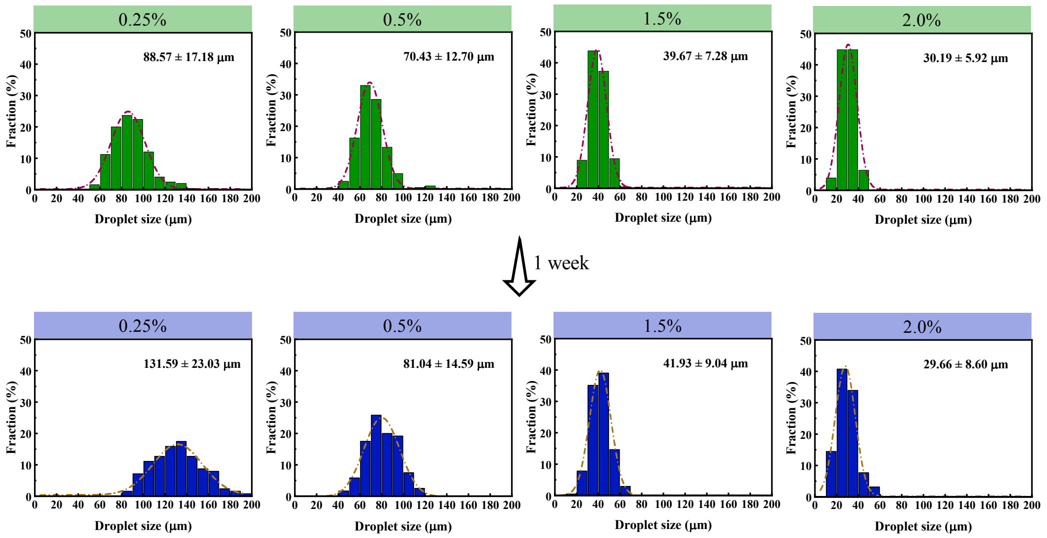

Figure S6. Droplet size distributions of Pickering emulsions (outer water droplet) prepared with 2.5% SA and various concentrations of hydrophobic SNPs during storage at room temperature.

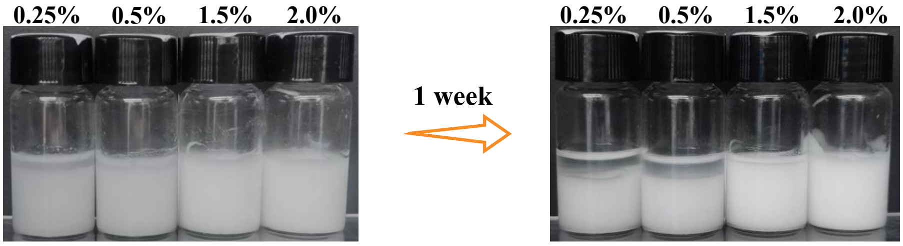

Figure S7. Appearance of Pickering emulsions prepared with 2.5% SA and various concentrations of hydrophobic SNPs during storage at room temperature.

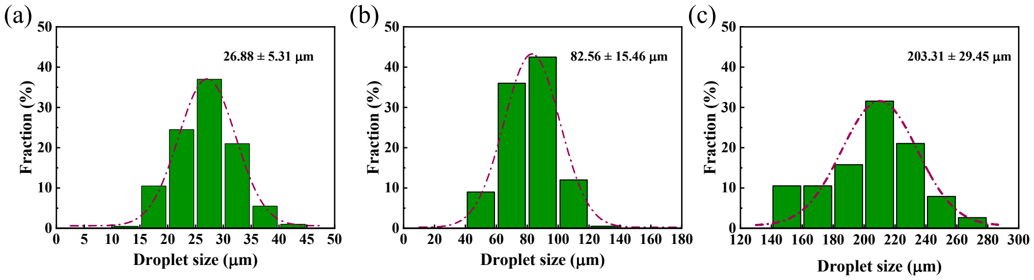

Figure S8. Droplet size distributions of emulsion (outer water droplet) prepared with 2.5% SA and 1% other commercially available SNPs with various hydrophobicity. (a) R816, (b) R812(s) and (c) R202.
